# Motor self-efficacy and physical education in school bullying

**DOI:** 10.3389/fpsyg.2024.1401801

**Published:** 2024-08-27

**Authors:** Carmen Galán-Arroyo, Elizabeth Flores-Ferro, Franklin Castillo-Retamal, Jorge Rojo-Ramos

**Affiliations:** ^1^University of Extremadura, Cáceres, Spain; ^2^Universidad Católica Silva Henríquez, Santiago, Chile; ^3^Universidad Católica del Maule, Talca, Chile

**Keywords:** bullying, motor self-efficacy, adolescents, school, health

## Abstract

In recent years, there has been a notable increase in bullying rates in school. Bullying is characterized as a repeated set of malicious actions by an individual or group toward another individual in an imbalanced power dynamic, resulting in physical, psychological, social, or material harm to the victim, with the intention of causing social exclusion. The scientific literature has investigated the factors that influence this type of behavior, environmental factors, such as school location and student age, as well as other factors, such as gender and level of physical fitness, also play an important role. This study examined the correlation between motor self-efficacy and bullying, considering that physical activity acts as a regulator in these cases. The Kolmogorov-Smirnov test was employed to assess the normality of the data distribution, and Spearman's Rho test was used to analyze the relationship between each dimension of the EBIPQ and E-AEM scores. The Cronbach's alpha was used to evaluate the reliability of each instrument. The results indicated a significant inverse correlation between the EBIP-Q and E-AEM in the variables of sex, educational stage and school environment. In conclusion, an inverse correlation was observed between motor self-efficacy and bullying, indicating that higher levels of motor self-efficacy are related to lower levels of bullying victimization. Motor self-efficacy could be used as a tool to reduce bullying situations at school. It would be important for government policies to encourage the practice of physical activity in all areas of life in order to improve motor self-efficacy and end bullying.

## 1 Introduction

Physical activity has become a tool for maintaining health because of the multiple benefits that it brings in different areas, such as psychological, physical, social, and physiological, increasing the quality of life of the person who practices it (Ai et al., 2021; Denche-Zamorano et al., 2022). International institutions, such as the World Health Organization (WHO), establish minimum recommendations for physical activity to maintain health according to the age and characteristics of the person; however, a high percentage of the world's population does not comply with these indications (World Health Organization, 2020, 2023). Specifically, in Spain, physical activity reports show that more than one-third of individuals aged between 15 and 69 years do not meet these recommendations (Ministerio de Sanidad, 2018). The rates of sedentary lifestyle and physical inactivity, in addition to entailing health problems, play a more important role in adolescents, as this is when physical changes occur and personality is built, establishing a critical period for the formation of habits and behaviors that can be maintained in the future In this sense, physical education is a means by which strategies can be developed to encourage physical activity and promote healthy social habits, with the aim of providing them with tools to improve their health. Physical activity and exercise also play important roles in the regulation of student behavior. On the one hand, it has a social factor, in which students must interact with each other in class to achieve the established objectives, with interaction based on respect, teamwork, and empathy; on the other hand, it acts as an emotional regulator (Welk and Schaben, 2004; Greco, 2021). It also has a strong influence on self-concept, which refers to a person's self-perceived knowledge and beliefs about themselves, encompassing elements such as physical, emotional and social aspects. Physical self-concept, is of utmost importance in the adolescent stage.

Bullying is defined as the set of actions repeated over time with the malicious character of a person or group toward another individual in a power imbalance relationship, in which the victim suffers physical, psychological, social, or material damage, with the aim of achieving social exclusion. It can occur in different contexts and environments, although it is more frequent in schools, where students spend more than 6 h a day and become one of the places where students spend more time. Therefore, it is important to make this environment a safe space and eliminate this type of behavior (Roland, 2002; Hamburger et al., 2011). This type of bullying has very negative consequences for the health of the victims, since in addition to multiple behavioral disorders, they may experience anxiety, depression, or suicidal ideas, as well as isolation and avoidance of attending school (Jimenez-Barbero et al., 2020), and their academic performance suffers. Despite the efforts of the educational community to prevent the spread of this type of behavior, recent studies in Spain show that school bullying is still more common than cyberbullying (Pichel et al., 2021), and the rate of students who suffer bullying is 30%, or about a third of students (Llorent et al., 2021), representing an important social problem. For the characterization of abusive and victimizing behaviors of bullying, the European Bullying Intervention Project Questionnaire (EBIP-Q; Brighi et al., 2012) was developed and subsequently validated in Spanish (Ortega-Ruiz et al., 2016) for the quantification of these cases. Studies related to bullying and the relationship with the practice of physical activity found that this improves factors such as self-esteem and perceived self-efficacy, in addition to the transmission of prosocial values, fundamental elements in the prevention of conflictive behaviors such as bullying and victimization of this same event (Pelegrín Muñoz et al., 2010), so that physical exercise is a fundamental tool in the prevention of these conflictive attitudes.

Researchers have studied the variables and factors involved in students' violent and abusive behaviors, which can act as protective factors for victims and preventive factors to avoid their development, with the aim of improving students' experiences in high schools and making them safe environments for all. Among these factors, physical activity has been studied, and it has been found that those who do not meet the minimum recommendations are more likely to suffer bullying (García-Hermoso et al., 2020). The consequences of this have also been studied, such as motor self-efficacy, defined as the perceived ability of each individual about his or her motor skills with respect to the challenges and obstacles that appear in everyday life and his or her ability to overcome them successfully (Hernández-Álvarez et al., 2011a). It has been shown that this variable affects academic performance, and specifically in the field of physical activity, acts as an emotional and behavioral regulator, decreasing the incidence of undesirable behaviors in individuals as they have greater motor auto efficacy (Pajares and Valiante, 2002; Greco, 2021). Studies in this area show that this variable can have protective and preventive effects on abusers and victims (Jimenez-Barbero et al., 2020). The Motor Self-Efficacy Scale (E-AEM) tool (Hernández-Álvarez et al., 2011a), enables the quantification of the level of motor self-efficacy perceived by students and identifies profiles and risk situations in this regard. Habits are determined and conditioned by the environment and the situation of the individual, such as the location of the school and the educational stage of the student.

For a better understanding of the context of the article, the sample chosen is in secondary education, divided between compulsory secondary education and baccalaureate. In Spain, the educational system consists of primary education, with students from 6 to 12 years of age; compulsory secondary education (CSE), consisting of four academic years from 12 to 16 years of age with a compulsory character where physical education is compulsory; and baccalaureate, consisting of two non-compulsory courses, where physical education is compulsory in the first course but not in the second.

For this reason, the aim of this study was to examine the associations between the motor self-efficacy scale and the EBIP-Q questionnaire, exploring the possible protective effects for victims and preventive effects on the appearance of this type of behavior related to the perception of motor skills, as well as exploring the significant differences between sexes, location of the school, and educational stage, and to investigate whether environmental factors intervene in these two variables.

## 2 Materials and methods

### 2.1 Participants

The sample consisted of 1,155 secondary school pupils from South Spain's both public and private schools. A convenience sample method was used for recruitment. Table 1 presents the participant's sociodemographic data.

**Table 1:** Participants' sociodemographic traits (*N* = 1,155).

**Variables**	**Categories**	***N***	**%**
School type	Public	869	75.2
	Private	286	24.8
Educational stage	CSE	877	75.9
	Baccalaureate	278	24.1
Sex	Male	564	48.8
	Female	591	51.2
School environment	Rural	368	31.9
	Urban	787	68.1

N, number; %, percentage.

### 2.2 Instruments

First, a preliminary questionnaire was created that asked questions on the students' type of school, educational stage, sex, and school environment they attended in order to gather sociodemographic information about the participating sample.

Then, the Spanish version of the European Bullying Intervention Project Questionnaire (EBIPQ) was applied (Hernández-Álvarez et al., 2011a). It consists of 14 items encompassed in two main dimensions, seven of which describe victimization-related features (Dimension 1) and seven of which relate to aggressiveness (Dimension 2). The items are behaviors like hitting, insulting, threatening, stealing, swearing, excluding, or spreading rumors, and they apply to both dimensions. Each item is designed in the format of a Likert scale, with a score between 0 and 4, where 0 denotes “Never” and 4 denotes “Always,” with a time span of the preceding 2 months. Finally, to gauge the level of motor self-efficacy in the school population, the Spanish version of the Motor Self-Efficacy Scale (E-AEM) was used (Hernández-Álvarez et al., 2011b). Ten questions on this test explain hypothetical situations that might occur when performing physical activity. A Likert-type scale is used, with values ranging from 1 to 4, with one signifying “totally disagree” and four signifying “totally agree.” A score is produced by applying the scale, with a minimum value of 10 denoting a low level of motor self-efficacy and a maximum value of 40 denoting the highest level of self-efficacy perception. According to the authors, Cronbach's alpha reliability was 0.89. Moreover, in this research, the victimization and abuser dimensions Cronbach's alpha values were 0.854 and 0.856, respectively, so they are defined as satisfactory. Additionally, the E-AEM result (Cronbach's Alpha = 0.907) could be regarded as excellent.

### 2.3 Procedure

Access was gained to the Department of Education and Employment's database in order to determine which schools provide physical education courses for Secondary Education through Baccalaureate (from 12 to 18 years of age). Inquiries on the availability of scheduling a researcher visit so that they may administer the questionnaire to the students who had their parents' informed consent were made via email to the physical education teachers working at these schools. They were also invited to respond to the inquiries using the same channel. The study's objectives, the parents' informed consent, and the instrument models used were all described in the email. If the instructors were willing to collaborate, they had to respond to the email by scheduling a time for a researcher to visit the school and, after getting parental consent, interview the students about bullying. Initial access to the questionnaire was provided to the students via a tablet, and each item was explained to them one at a time so they wouldn't have any doubts when responding. In order to prepare the data for a second researcher's later, blind analysis, the researchers sorted, cleansed, and anonymized the data after every questionnaire had been gathered.

A protocol adhering to the guidelines of the Declaration of Helsinki was approved by the Biosafety and Bioethics Committee of the University of Extremadura in Spain (Registration Code 71/2022).

### 2.4 Statistical analysis

In the present study, SPSS statistical software version 23 for MAC (IBM SPSS, Chicago, IL, USA) was used to process the data collected. A Kolmogorov-Smirnov test was first performed to examine the assumption of normality in the distribution of the continuous variables, and it was found that this assumption was not met; therefore, non-parametric statistical tests were used. To analyze the possible differences in the dimensions of the EBIPQ and in the total score of the E-AEM according to educational stage, sex, and school environment, the Mann-Whitney *U*-test was used. Likewise, Spearman's rho test was used to examine the relationship between variables. The thresholds proposed by Mondragón Barrera (2014) were adopted to interpret the correlation coefficients, ranging from 0.01 to 0.10 (low correlation), 0.11 to 0.50 (medium correlation), 0.51 to 0.75 (considerable correlation), 0.76 to 0.90 (very high correlation), and 0.91 to 1.00 (perfect correlation).

Finally, the Cronbach's alpha was used to examine the reliability of each instrument. The criteria set out by Nunnally and Bernstein (1994) were used as a reference to interpret the reliability test values as follows: <0.70 (low), 0.71–0.90 (satisfactory), and >0.91 (excellent).

## 3 Results

The scores obtained in both dimensions of the EBIPQ in terms of the categories belonging to the variables analyzed are shown in Table 2. When observing the educational stage, both CSE and baccalaureate students showed similar scores for victimization behaviors; however, significant differences were found for perpetration, with baccalaureate students expressing higher values. Likewise, sex seemed to influence both dimensions of the EBIPQ, showing different results. On the one hand, females were more related to victimization habits while males, on the other hand, reported higher perpetration habits. Finally, the school environment did not show differences in victimization behaviors; however, students from urban environments were designated more aggressive.

**Table 2:** EBIPQ by educational level, sex, and school environment.

	**Educational stage**			**Sex**			**School environment**		
**Item**	**CSE**	**Baccalaureate**	***p***	**Male**	**Female**	***p***	**Rural**	**Urban**	***p***
	**Me (IQR)**	**Me (IQR)**		**Me (IQR)**	**Me (IQR)**		**Me (IQR)**	**Me (IQR)**	
1) Victimization	1.86 (0.9)	1.86 (0.9)	0.80	1.71 (0.8)	1.86 (0.7)	<0.01^**^	1.86 (0.9)	1.86 (0.9)	0.28
2) Perpetration	1.43 (0.6)	1.50 (0.7)	<0.01^**^	1.43 (0.7)	1.43 (0.6)	<0.01^**^	1.29 (0.6)	1.43 (0.7)	<0.01^**^

Me, median value; IQR, interquartile range.

Differences are significant at ^**^p <0.01. Each score is obtained is based on a Likert scale (0–4): 0 is “Never” and 4 “Always.”

The total scores extracted from the participants and their differences according to the categories explored are listed in Table 3. High school students reported higher motor self-efficacy in the context of PE than their CSE peers. Similarly, the male students expressed higher levels of motor self-efficacy. In contrast, the educational environment of the school did not appear to be a determinant.

**Table 3:** Descriptive results of the E-AEM based on educational stage, sex, and school environment.

	**Educational stage**			**Sex**			**School environment**		
**E-AEM**	**CSE**	**Baccalaureate**	***p***	**Male**	**Female**	***p***	**Rural**	**Urban**	***p***
	**Me (IQR)**	**Me (IQR)**		**Me (IQR)**	**Me (IQR)**		**Me (IQR)**	**Me (IQR)**	
ΣE-AEM	31 (9)	32 (9)	<0.01^**^	33 (8)	29 (9)	<0.01^**^	32 (9)	31 (9)	0.32

Me, median value; IQR, interquartile range.

Differences are significant at ^**^p <0.01. Each score obtained is based on a Likert scale (1–4): 1 is “Totally disagree” and 4 “Totally agree.”

We also explored the possible correlations between the dimensions of the EBIPQ and the final E-AEM scores (Table 4) by comparing these associations in various categories of interest. In general, the dimension related to victimization showed mean, inverse, and significant correlations; on the other hand, no significant associations were found in aggressive behaviors, either at the general level or in any of the variables. For both CSE and baccalaureate students, the correlations in the first dimension were average, significant, and inverse, and were higher in the latter. As for sex, the victimization questions also showed mean, inverse, and significant associations, with male students expressing the highest coefficients. Finally, students from rural environments expressed greater relationships between motor self-efficacy and the victimization dimension than students from urban schools.

**Table 4:** Correlations between the EBIPQ and E-AEM scales by sex and educational level.

**EBIPQ dimensions**	**ΣE-AEM *ρ (p)***	**Educational stage**		**Sex**		**School environment**	
		**CSE Me (IQR)**	**Baccalaureate Me (IQR)**	**Male Me (IQR)**	**Female Me (IQR)**	**Rural Me (IQR)**	**Urban Me (IQR)**
1) Victimization	−0.12 (<0.01)^**^	−0.12 (<0.01)^**^	−0.14 (<0.01)^**^	−0.11 (0.01)^*^	−0.10 (0.03)^*^	−0.15 (<0.01)^**^	−0.11 (<0.01)^**^
2) Perpetration	−0.01 (0.63)	−0.04 (0.23)	0.05 (0.37)	−0.01 (0.80)	−0.07 (0.09)	−0.06 (0.29)	0.01 (0.86)

Differences are significant at ^**^p <0.01; ^*^p <0.05.

Finally, Table 5 shows the relationships between the scores obtained by the participants on both questionnaires. At the general level, bullying and motor self-efficacy were inversely correlated, low, and significant. When analyzing educational stage, only CSE students showed significant associations, which were inverse and medium. However, sex did not show significant correlations in any of the groups. Finally, self-efficacy and bullying were significantly related only in rural environments, and this association was characterized as medium and inverse.

**Table 5:** Correlations between E-AEM final score and EBIPQ score, according to educational stage, sex and school environment.

		**Educational stage**		**Sex**		**School environment**	
**Item**	**EBIPQ *ρ (p)***	**CSE**	**Baccalaureate**	**Male**	**Female**	**Rural**	**Urban**
		**Me (IQR)**	**Me (IQR)**	**Me (IQR)**	**Me (IQR)**	**Me (IQR)**	**Me (IQR)**
ΣE-AEM	−0.08 (<0.01)^**^	−0.11 (<0.01)^**^	−0.04 (0.54)	−0.07 (0.09)	−0.08 (0.06)	−0.12 (0.02)^*^	−0.06 (0.10)

Differences are significant at ^**^p <0.01; ^*^p <0.05.

## 4 Discussion

The main objective of this study was to explore the possible correlations between the EBIPQ and E-AEM scales, to determine whether the perceived motor efficacy of students could act as a protective or preventive factor of bullying, and to test the possible differences between sex, school location, and educational stage in which the students are in relation to these two tools.

First, we analyzed the significant differences that might exist between the three study variables related to EBIPQ scores: educational stage, sex, and school location. In the educational stage, those in the baccalaureate group had significantly higher scores than those in the CSE stage. In line with the results obtained in this work, the scientific literature supports these results, explaining that it may be due to the fact that older students publicly recognize that they are aggressors because of the social effect of recognizing power over others, feeling a kind of reward (Romera et al., 2016). Other studies also support these results by finding positive correlations between age and the perpetuation of bullying situations, establishing that older students tend to adopt this behavior to a greater extent than middle school students (Walters, 2021; Li et al., 2022). As a second variable, sex was analyzed in search of significant differences in the dimensions of the bullying questionnaire. The results show significant differences between sexes in both dimensions, with girls scoring higher in the victim dimension and boys scoring higher in the bully dimension. Although some studies find sex as a moderating variable in the effect on the aggressor dimension (Li et al., 2022), published studies do not find a consensus on which sex occupies each role the most. In articles that used the same tool, they found results similar to those of this study, in which boys had a higher prevalence in the aggressor area and girls in the victim area (González-Cabrera et al., 2020; Pichel et al., 2021). In contrast, other studies show that boys are more involved in both roles (Herrera-López et al., 2017; Feijóo et al., 2021; Obregon-Cuesta et al., 2022; Zhou et al., 2022). Finally, differences between rural and urban environments were studied, finding significant differences in the perpetration dimension, with those in urban schools scoring higher. In this sense, the scientific community also does not reach a consensus on the behavior of students depending on the environment in which the school is located, since some researchers find similar results to those found in this work (Cabrera et al., 2022) and other researchers find a higher degree of victimization in rural areas than in urban areas (Rodríguez-Álvarez et al., 2022), or even the opposite results to those found in this study, where students in rural environments are more involved in bullying than their counterparts in urban environments (Zhang et al., 2019). Owing to the scarcity of articles comparing these two variables, it is difficult to draw conclusions or explain this behavior. Further research is required in this regard.

In the statistical analyses of the E-AEM scale, significant differences were found with regard to educational stage, where high school students had a higher score, meaning that they had a higher motor self-perception than their CSE peers. In line with this finding, other authors agree with these results, stating that self-efficacy increases with age (Muñoz and García, 2013), although others report that the correlation is inverse, with those who were in earlier educational stages having higher self-efficacy due to the decrease in self-esteem in adolescence (Perea Chafé et al., 2016). Subsequently, statistically significant differences were found between sexes, with boys scoring higher than girls. This finding indicates that boys have a higher motor self-perception. The scientific literature finds similar results in this variable, explaining that motor self-efficacy is correlated with the practice of physical activity and enjoyment, finding that males generally perform more physical exercise than girls, so they have more development and motor self-perception (Çaglar, 2009; Hernández-Álvarez et al., 2011a; Chen et al., 2019; Ortiz Gómez, 2021).

Continuing with the results of the analysis of the correlations between the dimensions of the EBIPQ and the scores obtained in the E-AEM, we found significant inverse correlations in the victimization dimension, meaning that the higher the motor self-perception, the lower the score in the victim dimension, thus acting as a possible protective factor against bullying. This finding is in line with what has been found in other studies, which explain that high self-efficacy and motor skills are socially rewarded, and therefore, have social recognition and act as a protective agent (Kokkinos et al., 2015; Cao and Yang, 2018; Arcila-Arango et al., 2022). For the remaining variables, correlations of the same type were found in the victim dimension. In this sense, motor self-efficacy is a very important factor to consider in all contexts since it decreases the probability of bullying. The scientific literature finds physical activity and motor self-efficacy to be preventive tools for victims in all these contexts (Kokkinos et al., 2015; Cao and Yang, 2018; Benitez-Sillero et al., 2022). Taking the sum and global scores of both tools, the results show that the E-AEM has an inverse and significant correlation with the EBIPQ, demonstrating that high motor self-efficacy scores decrease bullying scores. Comparing the variables, significant correlations were only found in CSE, also with an inverse character, and in the rural environment, with correlations of the same nature. The scientific literature in this case provides an explanation for the correlation in CSE, where the score obtained in motor self-efficacy is higher because the levels of self-esteem are slightly higher and also act as an emotional regulator, in turn decreasing the occurrence of this type of conflictive behavior in the CSE stage, in addition to the fact that students perform more physical activity than students in higher grades (Waasdorp et al., 2019; Belcher et al., 2021).

### 4.1 Limitations and future lines

This study has certain limitations because it was conducted solely with students from the region of Extremadura, which means that the outcomes could be influenced by sociocultural factors. However, this presents an opportunity for further investigation in this field and opens up possibilities for future research. In addition, the data were gathered electronically, which has certain limitations (Nayak and Narayan, 2019). It would be worthwhile to investigate whether the results would have been the same if the questionnaires were delivered in a different format, if interviews were conducted, or if a qualitative study design had been utilized. In the future, it would be advantageous to expand the socio-demographic data of the participants to determine other variables that may clarify self-concept and self-esteem behaviors because one's perception of oneself could a risk factor for bullying, and to increase the sample size to include other communities at the national level.

### 4.2 Practical applications

The results obtained in this study are interesting. The fact that physical self-efficacy acts as a protective agent against bullying can be used as a tool to reduce the incidence of this phenomenon and improve school experience and coexistence. Physical activity is the means to improve physical self-efficacy, so physical education has the ideal means to promote prosocial values that improve this behavior, in addition to having the key to implementing pedagogical interventions in class to identify this type of behavior. Therefore, teachers have a scientific basis for optimizing this coexistence.

## 5 Conclusions

According to the study, physical self-efficacy is inversely related to bullying, so it can be used to reduce bullying situations at school to improve the coexistence of all students. Specifically, those who are in CSE and in rural areas have a higher risk of being a victim of bullying, so increasing physical self-efficacy, especially in these two contexts, would act as a protective agent against bullying, especially in the female sex. Therefore, it is important to apply these results in physical education in order to identify these situations and prevent them with the practice of physical activity.

## Data Availability

The raw data supporting the conclusions of this article will be made available by the authors, without undue reservation.
